# Excess Circulating Alternatively Activated Myeloid (M2) Cells Accelerate ALS Progression While Inhibiting Experimental Autoimmune Encephalomyelitis

**DOI:** 10.1371/journal.pone.0026921

**Published:** 2011-11-03

**Authors:** Ilan Vaknin, Gilad Kunis, Omer Miller, Oleg Butovsky, Shay Bukshpan, David R. Beers, Jenny S. Henkel, Eti Yoles, Stanley H. Appel, Michal Schwartz

**Affiliations:** 1 Department of Neurobiology, The Weizmann Institute of Science, Rehovot, Israel; 2 Department of Neurology, Methodist Neurological Institute, The Methodist Hospital Research Institute, The Methodist Hospital, Houston, Texas, United States of America; 3 NeuroQuest Ltd. Misgav Venture Accelerator, Misgav Business Park, Misgav, Israel; National Institutes of Health, United States of America

## Abstract

**Background:**

Circulating immune cells including autoreactive T cells and monocytes have been documented as key players in maintaining, protecting and repairing the central nervous system (CNS) in health and disease. Here, we hypothesized that neurodegenerative diseases might be associated, similarly to tumors, with increased levels of circulating peripheral myeloid derived suppressor cells (MDSCs), representing a subset of suppressor cells that often expand under pathological conditions and inhibit possible recruitment of helper T cells needed for fighting off the disease.

**Methods and Findings:**

We tested this working hypothesis in amyotrophic lateral sclerosis (ALS) and its mouse model, which are characterized by a rapid progression once clinical symptoms are evident. Adaptive transfer of alternatively activated myeloid (M2) cells, which homed to the spleen and exhibited immune suppressive activity in G93A mutant superoxide dismutase-1 (mSOD1) mice at a stage before emergence of disease symptoms, resulted in earlier appearance of disease symptoms and shorter life expectancy. The same protocol mitigated the inflammation-induced disease model of multiple sclerosis, experimental autoimmune encephalomyelitis (EAE), which requires circulating T cells for disease induction. Analysis of whole peripheral blood samples obtained from 28 patients suffering from sporadic ALS (sALS), revealed a two-fold increase in the percentage of circulating MDSCs (LIN^−/Low^HLA-DR^−^CD33^+^) compared to controls.

**Conclusions:**

Taken together, these results emphasize the distinct requirements for fighting the inflammatory neurodegenerative disease, multiple sclerosis, and the neurodegenerative disease, ALS, though both share a local inflammatory component. Moreover, the increased levels of circulating MDSCs in ALS patients indicates the operation of systemic mechanisms that might lead to an impairment of T cell reactivity needed to overcome the disease conditions within the CNS. This high level of suppressive immune cells might represent a risk factor and a novel target for therapeutic intervention in ALS at least at the early stage.

## Introduction

Amyotrophic lateral sclerosis (ALS), commonly known as Lou Gehrig's disease, is an adult-onset neurodegenerative disease that selectively destroys upper and lower motoneurons, resulting in muscle weakness and atrophy, progressive paralysis, respiratory failure, and eventually death within 4–6 years [1], [2], [3]. The role of peripheral immune cells in this devastating disease has only been recently appreciated. Several studies suggest that boosting peripheral immunity, rather than suppressing it, might allow for better recruitment of circulating immune cells to sites of neurological damage, thereby ameliorating local toxic inflammatory effects and possibly augmenting protective responses adjacent to injured neurons [4], [5], [6], [7]. More recently, studies investigating the systemic immune system of sALS patients revealed changes in peripheral blood T cells [8], [9], [10], monocytes, and in chemokine receptor 2 (CCR2) expression levels on monocytes [9], [11], suggesting an important role for elements of the immune system in ALS patients.

Myeloid-derived suppressor cells (MDSCs) constitute a unique component of the immune system that regulate immune responses in healthy individuals and in various diseases [12]. Yet, like any immunoregulatory cell, if their levels become excessive, their effect may be detrimental rather than helpful. For example, in cancer, peripheral immunosuppressive cells may increase over the course of tumor development [13], [14], [15], contributing to an escape from immune system surveillance and increased tumor proliferation. Our hypothesis is that such a dysregulation of the immune response may also play an important role in neurodegenerative diseases such as sALS, and thus could be a disease risk factor. In mice, MDSCs are characterized by the co-expression of the myeloid-cell lineage differentiation antigen Gr1 and CD11b. Normal mouse bone marrow contains 20–30% of cells with this phenotype, but these cells make up only a small proportion (2–4%) of spleen cells and are absent from the lymph nodes [12]. Another population of myeloid cells that exhibit suppressive activity is the alternatively activated macrophages, designated M2 macrophages. These cells share many characteristics of tumor-associated macrophages (TAMs), which help the tumor to escape from immune-mediated killing [16]. In humans, MDSCs are most commonly defined as cells that express the common myeloid marker CD33 but lack the expression of markers of mature myeloid and lymphoid cells, and of the MHC class II molecule HLA-DR (CD33^+^LIN^–^HLA-DR^–^).

Here, we show that systemic administration of IL-4 treated bone marrow-derived myeloid cells (BMDM) to G93A mutant superoxide dismutase-1 (mSOD1) mice (ALS mice) before emergence of disease symptoms resulted in earlier appearance of disease symptoms and shorter life expectancy, while the same protocol mitigated the inflammation-induced disease model of multiple sclerosis, experimental autoimmune encephalomyelitis (EAE). Analysis in patients suffering from ALS revealed a two-fold increase in the percentage of the circulating subset of MDSCs (LIN^−/Low^HLA-DR^−^CD33^+^) in sporadic ALS (sALS) patients compared to controls.

## Results

**Figure 1:**
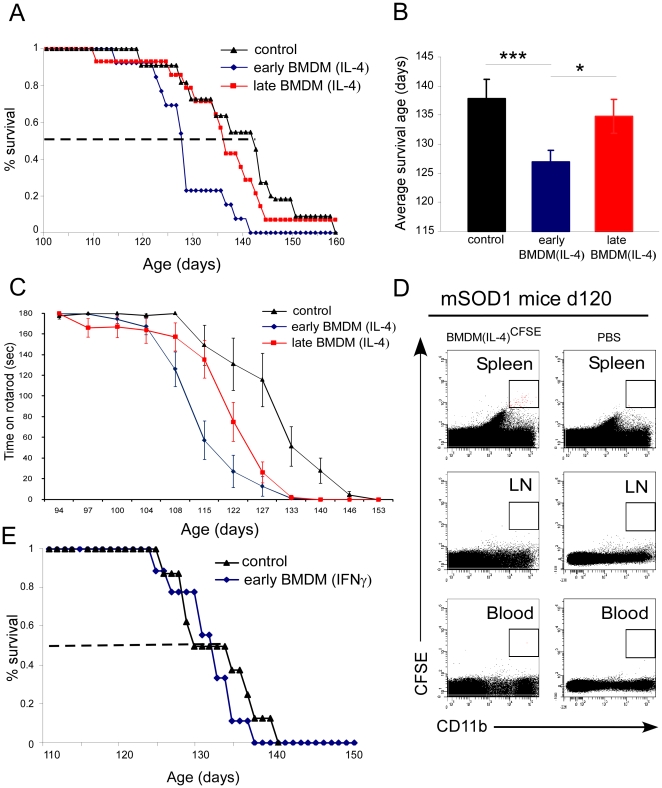
Shorter survival times and earlier appearance of disease symptoms following repeated administration of IL-4 treated BMDM. (**A–B**) Male mSOD1 mice on a B6/SJL genetic background receiving repeated injections of IL-4-activated BMDM starting from day 70 (early BMDM_IL-4_) had a shorter survival time (127±2 days, n = 13) than non-injected mSOD1 control littermates (138±3 days, n = 11; P = 0.003, log-rank-sum test). Survival of male mSOD1 mice receiving repeated IL-4-activated BMDM starting from day 90 (late BMDM_IL-4_) was similar to control mSOD1 littermates (135±3 days, n = 14; P = 0.515). (**C**) Effects of early and late treatment with IL-4-activated BMDM on Rotarod performance compared to control mice. Rotarod performance in mice with early treatment declined at most time points, between 109 and 150 days of age, compared with untreated control mSOD1 littermates (P<0.0001, one-way ANOVA; each pair Student's t test, *early vs. control; § late vs. control; #early vs. late). (**D**) Survival of male mSOD1 mice receiving repeated injections of IFNγ activated BMDM starting from day 70 (early BMDM_IFNγ_) was similar (130±1.4 days, n = 9) to untreated control mSOD1 littermates (131±1.9 days, n = 8).

To test our working hypothesis that systemic myeloid-derived suppressor cells might have a role in ALS, we first examined the potential ability of such cells to affect the kinetics of disease onset and progression in ALS mice. Suppressive activity of myeloid-derived suppressor cells in humans was shown to be mediated by the enzyme arginase 1 (Arg1), which can be induced in myeloid-derived cells by IL-4. Therefore, we used IL-4 activated BMDM cells in our attempts to modify the disease course. In order to examine the impact of these cells on both disease onset and progression, we repeatedly injected mSOD1 mice intravenously (i.v) with IL-4-activated BMDM from day 70 onward, every fifth day (early BMDM_IL-4_). As controls, we used a group of non-injected mSOD1 mice (control), a group of mSOD1 mice that received the same cells starting on day 90 (late BMDM_IL-4_), and a group of mSOD1 mice that received IFNγ-activated BMDM from day 70 onward (early BMDM_IFNγ_). Survival of mSOD1 mice receiving early treatment with IL-4-activated BMDM cells was shorter compared to control, untreated mice (P = 0.003, log-rank-sum test), and compared to mSOD1 mice receiving late treatment with the same cells (P = 0.012, log-rank-sum test; **Figure 1A**–**B**). The median survival of mSOD1 mice receiving early treatment with IL-4 activated BMDM was 127d, compared to 141d for control mice and 135d for mice receiving late treatment with IL-4 activated BMDM cells. Similarly, early treatment of mSOD1 mice with IL-4-activated BMDM cells resulted in earlier appearance of clinical symptoms compared to mice receiving late treatment and to control untreated mice, as monitored by a decline in Rotarod performance (P<0.0001, 2-factor repeated measures ANOVA; **Figure 1C** **)**. Although the late administration of IL-4 activated BMDM cells to mSOD1 did not significantly affect mouse survival compared to control animals, a significant reduction in motor ability was observed starting on day 127 through day 140, as monitored by Rotarod performance (P<0.001, Student's t test, **Figure 1C** **)**.

**Figure 2:**
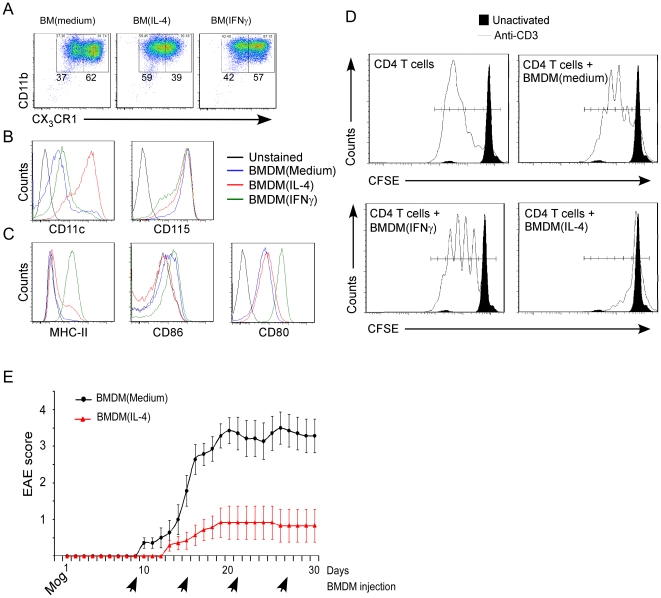
IL-4 activated BMDM cells inhibit CD4 T cell activation and ameliorate EAE. Bone marrow derived myeloid cells (BMDM) were cultured from *Cx_3_cr1* ^GFP/+^ mice (B6.129P- *Cx_3_cr1*tm1Litt/J, in which one of the *CX_3_CR1* chemokine receptor alleles was replaced with a gene encoding GFP [green fluorescent protein]), and treated with IL-4 or IFNγ as described in Methods. (**A**) Acquired cells were first gated for live cells and the fraction of cells expressing CD11b and *Cx_3_cr1* ^GFP+^ (high and low) was determined. (**B**) Histograms showing the expression levels of CD11c and CD115 within the CD11b^+^ *Cx_3_cr1* ^GFP+^ cell population are shown. (**C**) Histograms showing the expression levels of MHCII, CD80 and CD86 within D11b+ *Cx_3_cr1* ^GFP+^ cells are shown. (**D**) Effect of BMDM on T cell proliferation *in vitro*. Total splenocytes were labeled with CFSE, stimulated with or without anti-CD3 (1 µg/ml), and co-cultured with IL-4 or IFNγ– activated BMDM cells. On day 3, cells were harvested and CFSE dilution in CD4 T cells (CD4^+^TCRβ^+^) was measured by flow cytometry. (**E**) Chronic EAE was induced in C57BL/6J mice. EAE scores in mice injected with IL-4-activated BMDM (MOG+BMDM_IL-4_) (n = 6) or with nonactivated BMDM (MOG+ BMDM_Medium_) (n = 7), administered every 6^th^ day starting 9d after MOG vaccination are shown. Intravenously injected IL-4-activated BMDM cells significantly improved clinical features in mice with chronic EAE compared to mice treated with non-activated BMDM cells (2-factor repeated measures ANOVA; P<0.001; **P<0.01, ***P<0.001, Student's t test).

To monitor the in vivo homing of the injected IL-4-activated BMDM cells, they were labeled with CFSE prior to injection. CFSE-labeled IL-4 activated BMDM were detected in the spleens of mSOD1 mice, while no cells were detected in the blood and peripheral lymph nodes (LN) or CNS (**Figure 1D** **)**. Importantly, the survival of mSOD1 mice receiving repeated injections of IFNγ-activated BMDM starting from day 70 (early BMDM_IFNγ_) was not affected, and was similar to that of untreated control mSOD1 littermates (**Figure 1E** **).** Furthermore, early treatment of mSOD1 mice with IFNγ-activated BMDM did not affect their motor ability compared to control mice, as monitored by Rotarod performance (data not shown). To evaluate the immune markers and the suppressive function of IL-4-activated BMDM versus IFNγ-activated BMDM, also known as M2 and M1 myeloid cells, respectively, we further characterized the isolated BMDM cells from *Cx3cr1* ^GFP/+^ mice [17] following their treatment with IL-4 or with IFNγ, as described in Methods. Immunophenotyping of the cytokine-treated and untreated BMDM cells revealed that they were positive (>98%) for CD11b, CD115, and CX3C chemokine receptor 1 (CX3CR1) also known as the fractalkine receptor (**Figure 2A**–**B**). Measurement of activation markers revealed that IL-4 activation resulted in higher expression of CD11c and lower expression of MHCII, CD80 and CD86 compared to IFNγ treated cells (**Figure 2C**). As shown in **Figure 2D**, IL-4 activated cells efficiently inhibited CD4 T cell antigen receptor-mediated proliferation, while IFNγ-activated (M1) myeloid cells had only a slight effect on CD4 proliferation.

To ensure the in vivo suppressive potential of systemic administration of the IL-4-activated BMDM, we used a mouse model of multiple sclerosis, which represents an inflammatory T-cell mediated CNS disease that can be arrested by systemic anti-inflammatory treatment. Chronic EAE was induced in C57BL/6J adult mice, and clinical signs were evaluated daily as described in Methods. Animals were either injected (every fifth day) with non-activated BMDM, or with IL-4-activated BMDM starting 9 days after MOG vaccination. Systemic i.v administration of IL-4-activated BMDM cells significantly reduced the severity score of EAE compared to animals treated with non-activated BMDM cells (2-factor repeated measures ANOVA; P<0.001; **Figure 2E**). Thus, the immunosuppressive phenotype of the IL-4-activated BMDM was verified by their suppressive effect in an inflammatory disease model. These experiments showed that while elevated levels of IL-4-activated BMDM had a beneficial impact on the EAE model, in which their effect is manifested by inhibition of the systemic autoimmune attack on the CNS, they had a negative impact on mSOD1 mice, resulting in an earlier onset of the disease symptoms and a shorter life expectancy.

**Figure 3:**
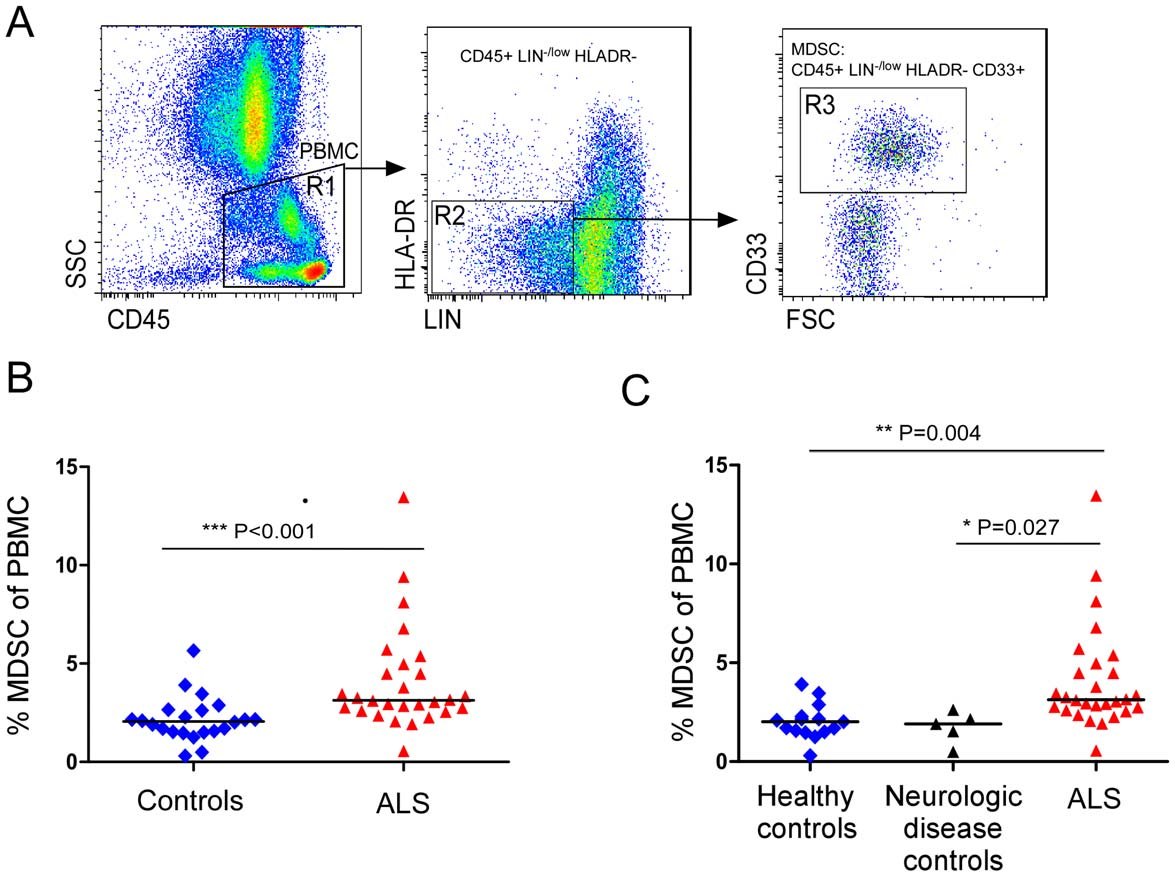
Higher percentage of circulating MDSCs in sALS patients relative to controls. (**A**) Representative flow diagrams from one ALS patient are shown. Acquired cells were first gated (R1) based on the expression of CD45; this population was comprised of total peripheral mononuclear cells (PBMCs). Acquired cells were then gated (R2) by selecting Lin ^−/Low^, HLA-DR negative cells. Within this population, the fraction of cells expressing CD33 was determined (R3). The MDSC population was defined as Lin^−/Low^, HLA DR negative, and CD33 positive cells. MDSC percentage was calculated as the percentage of total CD45 positive PBMCs in the whole blood samples. (**B**) The percentage of circulating MDSC was significantly higher in ALS patients compared with control donors (*p* = 0.001); bar represents the median. (**C**) The proportion of MDSC in ALS patients (n = 28) was increased compared to both healthy controls (n = 15; *p* = 0.004) and to neurologic disease controls (n = 5; *p* = 0.027).

To assess the relevance of such a cell population to ALS patients, we examined whether ALS patients have higher levels of myeloid-derived cells that could contribute to suppressor effect in the circulation. To this end, we first collected whole peripheral blood samples from 15 patients (mean age±SD, 55±10 years, n = 15 11M, 4F) with the clinical diagnosis of definite ALS, and from 10 healthy volunteers (52±10 years, n = 10, 6M, 4F) and analyzed them by flow cytometry for the presence of circulating myeloid-derived cells reminiscent of MDSCs, monocytes, granulocytes and T cells. The MDSCs in the patients were defined as lineage negative/low (LIN^−/Low^), HLA-DR negative, CD33 positive cells, based on previously described parameters [13]. A higher percentage of circulating MDSCs out of total PBMCs was observed in sALS patients relative to healthy volunteers (mean±SD, 3.6±1.9 vs. 2.04%±1, respectively; p = 0.02). An increased percentage of granulocytes from total leukocytes were also found in sALS relative to healthy controls (54.7±8.7 vs. 46%±8.9, respectively; *p* = 0.025). Based on these initial results, we analyzed additional patients; altogether, our studies included 28 ALS patients and 22 control volunteers including 7 control patients suffering from other diseases (**Table 1**). Representative flow diagrams summarizing the phenotypic analysis of MDSCs are shown in **Figure 3A**. MDSCs were defined as LIN^−/Low^, HLA-DR negative, CD33 positive cells (R3) out of gated PBMC. A 2-fold increase in the percentage of circulating MDSCs in sALS patients relative to all control subjects was observed (4.1±2.6 vs. 2.1%±1.1, respectively; *p* = 0.0013; **Figure 3B**). The higher percentage of MDSCs was observed in sALS patients when we compared them to healthy volunteers, or to patients with other neurologic diseases (*p* = 0.004, *p* = 0.027, respectively; **Figure 3C**). Further analysis of MDSCs revealed heterogeneous cell morphology, suggesting that this population consists of two major cell types (data not shown).

**Table 1:** Characteristics of sALS patients and control subjects.

Characteristics of sALS patients	sALS patients (n = 28)
Age^a^	56.5±9.7 years (37–73)
Sex	M = 19; F = 9
Location of onset	Spinal = 79%; bulbar = 21%
ALSFRS-R score (0–48)^b^	28 (6–45)
Characteristics of controls	Controls (n = 22)
Age^a^	58.9±12 years (38–78)
Sex	M = 10; F = 12
Healthy controls	n = 15
Neurologic disease controls	n = 5; 3 diagnosed with CIDP, 1 diagnosed with cervical myelopathy, 1 diagnosed with multi-system atrophy
Non-neurologic disease controls	n = 2; 1 with CVID, 1with COPD

a Mean±SD (age range, years)

b Median (range)

Chronic Inflammatory Demyelinating Polyneuropathy (CIDP).

Common Variable Immuno-deficiency (CVID).

Chronic Obstructive Pulmonary Disease (COPD).

To evaluate whether the levels of MDSCs could serve as a potential risk factor for ALS, an analysis of receiver operating characteristics (ROC, specificity vs. sensitivity) was performed. The analysis revealed that the optimal cutoff, which represented the percentage of MDSCs out of PBMCs, to distinguish sALS patients from all control subjects was 2.28 percent. This cutoff value distinguished sALS patients from all control subjects with 82% accuracy, 86% sensitivity and 73% specificity (**Figure S1A**). Using an identical cutoff, sALS patients were differentiated from selected control subjects (n = 20), excluding two patients diagnosed with severe immunosuppression, with 86% accuracy, 86% sensitivity and 80% specificity (**Figure S1B**). There was no correlation between the levels of MDSC and the age of each sALS patient, nor with their ALSFRS score as a measure of disease severity / stage (data not shown). A smaller group of sALS patients (n = 9) was assessed for MDSC levels 3 months after the initial measurements. The individual patient characteristics are summarized along with a representative flow diagram summarizing the phenotypic analysis of MDSCs (**Figure S2A**–**B**). Although this period was not sufficient to expect to see any change among a population of patients who were not in synchrony in terms of disease stage, ALSFRS score analysis indicated a decrease in patient score at the second clinic visit compared with the first visit (p = 0.05, paired t test), with a trend associated with higher MDSC levels (p = 0.096, **Figure S2C**).

## Discussion

In the present study, we showed in a mouse model of ALS that disease course was exacerbated following systemic adoptive transfer of IL-4-activated BMDM (M2 myeloid cells), which suppress peripheral T cell activity. Such treatment in an animal model of an inflammation-induced neurodegenerative disease, EAE, provided a beneficial effect. These results strongly support the contention that although these two CNS pathologies share local inflammation, they respond in an opposite manner to systemic anti-inflammatory treatment. Moreover, the negative effect in ALS mice obtained with BMDM activated by IL-4, which is known to be a strong inducer of Arginase 1, a characteristic feature of MDSCs, was apparently consistent with the observed elevation in the proportion of MDSCs in the peripheral blood of ALS patients. Such MDSCs in cancer patients are known for their ability to inhibit T cell activity [13], [14]. Further studies are necessary to confirm the in vitro suppressive function of ALS patient's MDSCs.

ALS is a neurodegenerative disease that selectively affects motor neurons in the CNS, leading to bulbar, respiratory, and limb weakness. The mouse model we used (mSODG93A) is the most common model in the field of ALS for pre-clinical research. In the present study, we evaluated the in vivo effect of excess peripheral myeloid-derived anti-inflammatory cells on a disease progression. The animal model of multiple sclerosis, EAE, was chosen here as a control for the in vivo immunosuppressive effect of the anti-inflammatory (M2) myeloid-derived cells. Multiple sclerosis, unlike ALS, is a chronic inflammatory disease in its etiology, which leads to cumulative and irreversible CNS damage [18]. We expected that if these myeloid-derived suppressor cells (M2) would show a suppressive effect in EAE, the outcome in ALS could be reliably attributed to T cell immunosuppression in the peripheral immune organs. A previous study reported an accumulation of Arg-I+-MDSCs in the spinal cord during EAE; these cells were largely restricted to the demyelinating plaque. The presence and density of Arg-I+-cells, and the proportion of apoptotic but not proliferative T cells, were correlated with the time course of EAE; cell numbers peaked in parallel with the clinical score, decreased significantly during the remitting phase, and completely disappeared during the chronic phase [19].

It is accepted that ALS, although affecting motor neurons, non-neuronal cells play critical roles in disease progression [4], [20], [21], [22]. The present study introduces an additional cell population potentially involved in disease regulation, thereby extending the scope of the players that have been identified in this disease beyond the CNS, which includes circulating T cells, NKT cells and monocytes [4], [21], [22], [23], [24]. In a model of acute spinal cord injury, circulating T cells facilitate recruitment of monocytes to the injured CNS that locally suppress the neurotoxic responses of microglia [4]. Moreover, in both acute spinal cord injury and an ALS mouse model, CD4^+^ T cell deficiency accelerates the degenerative process [4], [22], [25], [26]. Our present results demonstrating that the increased levels of circulating IL-4-activated anti-inflammatory BMDM that homed to the spleen (a peripheral lymphoid organ) and presumably had a negative effect on the availability of T cells, support the contention that circulating T cells are needed for protecting the motor neurons, either by their direct recruitment to the CNS or by facilitating infiltration of monocytes that locally suppress the microglia. A previous study suggested an increase expression of F/480 and Gr-1 in the perifollicular area of spleen from SOD1 Tg mice compare to age-matched wild-type controls [26]. The fact that late administration of anti-inflammatory myeloid-derived cells to mSOD1 did not significantly affect their survival compared to control mice, while the survival times of mSOD1 mice receiving these cells at an early stage was significantly shorter (by 14 days), suggests that either the disease is too advanced to be affected or that the systemic immune response has already been deteriorated at this stage, and thus any further addition of cells is unlikely to have significant effect [26]. Thus, it appears that in the advanced stage of the disease, the capacity of the remaining circulating immune cells in these mice to support the CNS is diminished, which might explain the lack of any further suppression by the injected suppressor cells. Collectively, the fact that mSOD1 mice that received IL-4 activated BMDM showed earlier disease symptoms, suggests that systemic immune suppression found in patients could be viewed as co-morbidity factor in ALS. In cancer, such cells have been linked with disease progression via a mechanism induced by the tumor itself as a means of escaping from immune surveillance and immune-mediated elimination [20], [27]. A recent study suggested up-regulation of a T cell co-stimulation pathway and a key regulator of immune responses in ALS mice and patients [28], possibly in response to increased MDSC levels. Importantly, a significant reduction in the number of thymic progenitor-cells and abnormal thymic histology was observed in ALS mice [29]. Additionally, a reduction in blood levels of T cell receptor rearrangement excision circles (sjTRECs), as a measure of thymic output, and a restricted T cell repertoire accompanied by an increase in pro-apoptotic markers in ALS patients were demonstrated [29].

As MDSCs are one of the main circulating immunosuppressive factors in cancer and other pathological conditions, several different therapeutic strategies that target these cells are under investigation. Some of the strategies include promoting myeloid cell differentiation into non immunosuppressive lineages, inhibiting their expansion, neutralizing their products, or elimination of this population [12]. In summary, the finding from this study uncover a possible risk factor and a novel target for therapeutic intervention in ALS, while the same cell population limits the inflammatory damage in a mouse model of MS. Interference with the suppressive activity of MDSCs has been shown to restore normal immune function in tumor-bearing hosts. Thus for example, L-arginine metabolism in MDSCs might be an excellent molecular target for a novel class of immunoregulatory compounds in early ALS to restore immune function and increase the efficacy of any immune-based therapy.

## Materials and Methods

### Ethics Statement

This study was conducted according to the principles expressed in the Declaration of Helsinki. All subjects participating in this study signed an informed consent form approved by the Methodist Hospital Institutional Review Board (IRB0807-0139). All animal work was conducted according to national and international guidelines and approved by Weizmann Institute's Animal Care and Use Committee (IACUCU; permission number: 03680806-1 and 03740707-2).

### Animals

Inbred adult male C57BL/6J mice (8−10 weeks old), ALS mice [B6SJL-Tg (SOD1-G93A)1Gur/J; Jackson Laboratories, ME], and their WT B6SJL littermates were used. Animals with ALS were anesthetized when no longer able to right themselves within 30 seconds of being placed on their sides. Cx3cr1GFP/+ mice were used as donors for BMDM cells (B6.129P- Cx3cr1tm1Litt/J, in which one of the CX3CR1 chemokine receptor alleles is replaced with a gene encoding GFP [green fluorescent protein]).

### Patients and control individuals

Patients were enrolled from Methodist Hospital Muscular Dystrophy/ALS clinic. ALS patients were not being treated with riluzole during the study, and all provided written informed consent. Peripheral whole blood was drawn from 28 sALS patients (mean age 56; range 37–73) diagnosed with definite or probable sporadic ALS according to revised El Escorial criteria of the World Federation of Neurology [30] in order to identify biological markers by flow cytometry. Additional peripheral blood samples were donated by 22 control volunteers (mean age 59; range 38–78) after giving written informed consent. The control volunteers included 15 normal healthy volunteers without histories of infectious diseases or other disorders, 5 neurologic disease controls, and 2 non-neurologic disease controls. From each participant, 25 ml of venous blood was collected in K2 EDTA-lavender top tubes (BD, Franklin Lakes, NJ). sALS patient and control group characteristics are detailed in **Table 1**.

### Antibodies and flow cytometry

Antibodies used for cell surface labeling of mouse BM myeloid cells included PE or PercP-Cy5.5 conjugated CD11b (clone M1/70; BioLegend), PercP conjugated CD86 (clone GL-1; BioLegend), APC conjugated CD11c (clone N418; eBioscience), APC conjugated CD80 (clone 16-10A1; eBioscience), PE conjugated MHC-II (clone N/MR-4; eBioscience) and PE conjugated CD115 (clone AFS98; eBioscience). Flow cytometry was performed on each sample using a BD Biosciences LSRII flow cytometer, and the acquired data were analyzed using FlowJo software.

Antibodies used for cell surface labeling of human whole blood leukocytes included FITC conjugated Lineage Cocktail 1 (LIN-1)(CD3 clone SK7, CD14 clone MφP9, CD16 clone 3G8, CD19 clone SJ25C1, CD20 clone L27, CD56 clone NCAM16.2; BD Biosciences), HLA-DR (PE-conjugated clone TU36; BD Biosciences), CD45 (PercP--conjugated clone 2D1; BD Biosciences), CD33 (APC conjugated LEU-M9; BD Biosciences), CD3 (FITC-conjugated clone SK3; BD Biosciences), CD8 (PE-conjugated clone SK1; BD Biosciences), CD4 (APC-conjugated clone SK7; BD Biosciences), and appropriate isotype-matched control antibodies (BD Biosciences). Staining was performed on fresh venous blood collected in EDTA-coated vacutainer tubes (BD Biosciences). Briefly, 100 µL of whole blood was mixed with 5 µL of each antibody used, in a BD Falcon™ round-bottom tube. Samples were incubated at room temperature for 30 minutes; then, each sample was mixed with 2 mL of 1x erythrocyte lysis buffer (BD Biosciences) and incubated for an additional 15 minutes to remove red blood cells. Samples were spun at 1500 rpm for 5 minutes in a refrigerated centrifuge, supernatants removed and washed with FACS buffer (1% FBS in PBS, 0.09% sodium azide). Cell pellets were then resuspended in 300 µL of FACS buffer. Flow cytometry was performed on each sample using a BD Biosciences LSRII flow cytometer. Acquired data were analyzed using FlowJo software.

### CFSE inhibition assay

Total splenocytes (1*10^5^ per well) were labeled with CFSE (Molecular Probes) (2.5 µM in PBS for 10 min). Labeled cells were washed twice to remove unbound CFSE, incubated with 1 µg/ml anti-CD3 (145-2C11, eBioscience) and co-cultured in complete medium for 72 hrs with untreated or with IL-4 or IFNγ–activated BMDM cells (5x10^4^ per well). CFSE dilution in CD4 T cells (CD4^+^TCRβ^+^) was measured by flow cytometry.

### Preparation of bone marrow-derived myeloid cells

Bone marrow cells were harvested from both the hindlimbs (tibia and femur) and the forelimbs (humerus) of WT mice (8−10 weeks old) by flushing the bones with Dulbecco's phosphate-buffered saline (PBS; Sigma-Aldrich) under aseptic conditions. Cells were collected and centrifuged (10 min, 1000 rpm, 4°C), resuspended, and then seeded (7 × 10^6^ cells) in 10 ml of culture medium [RPMI-1640 medium supplemented with 10% fetal calf serum, L-glutamine (1 mM), sodium pyruvate (1 mM), β-mercaptoethanol (50 µM), penicillin (100 U/ml) and streptomycin (100 U/ml)], and cultured at 37°C/5% CO_2_ in 75-cm^2^ tissue-culture flasks previously coated with poly-D-lysine (PDL) in borate buffer (pH 8.4). Culture medium and floating cells were discarded every 4^th^ day, and fresh medium was added. After 10 days of incubation, nonadherent cells were washed out, and the flasks with remaining adherent cells were refilled with fresh medium. The cells were then left untreated or were treated for 2 days with 10 ng/ml of recombinant mouse IL-4 (endotoxin<0.1 ng perµg, R&D Systems) or IFN-γ (endotoxin<0.1 ng perµg, R&D Systems). On the day of injection, the adherent cells were washed twice with cold PBS and placed on ice for 10 min; then, the cells were removed using a cell scraper, counted and injected (2×10^5^ cells in 100 µl of PBS) to the tail vein. Purity of the cells was assessed by FACS analysis after staining with antibodies (Abs) against Mac-1 (CD11b) (Pharmingen), and was found to be >98%. For detection of BMDM cells following i.v injection (Fig. 2D), cells were labeled with CFSE prior to injection: 2x10^6^ BMDM cells (20x10^6^ cells/mL) were incubated in PBS (without Ca2+/Mg2+) supplemented with 5 µM CFSE (Molecular Probes) for 8 min at 25°C. Following incubation, the cells were washed three times with RPMI containing 8% fetal calf serum (FCS). Then, 2x10^6^ CFSE-labeled BMDM in 100 µl PBS were injected into the tail vein of mSOD1 mice (120d, n = 3). After 24 hr, blood, spleen and peripheral LN were removed, and CD11b^+^ CFSE^+^ cells were analyzed by LSRII flow cytometer.

### Assessment of mouse motor ability

Mice were inspected routinely for general condition. From 60 d of age onward, mice were assessed for motor dysfunction using the Rotarod apparatus (Jones and Roberts 7650, Ugo Basile, Italy). Animals were placed on an accelerating rod, and time required for each mouse to fall from the rod was recorded. Out of three trails, the longest time taken for an animal to fall from the rod was recorded and used for analysis. A cut-off time point of 180 sec was defined, and mice remaining on the rod for at least this period of time were deemed asymptomatic.

### Induction and evaluation of acute and chronic EAE

To induce chronic EAE, adult male C57BL/6J mice were injected s.c. with 200 µg (300 µl) of MOG_35–55_ (Sigma-Aldrich) in incomplete Freund's adjuvant containing 2.5 mg/ml *M. tuberculosis* (strain H37Ra; BD Diagnostics). Pertussis toxin (500 ng; Sigma-Aldrich) was injected on the day of the immunization, and again 2 days later. Clinical signs were evaluated daily in a blinded fashion by at least two investigators, and an EAE score was assigned (0, healthy; 1, limb tail paralysis; 2, ataxia and/or paresis of hind limbs; 3, paralysis of hind limbs and/or paresis of forelimbs; 4, tetraparalysis; 5, moribund state or death).

### Statistical analyses

The SPSS and SAS statistical packages were used to analyze all data. Comparisons between sALS patient and control lymphocytes, monocytes and MDSC frequencies were analyzed using a two tailed Student's unpaired t test. The same analysis was also used to assess the influence of age differences. Comparisons of ALSFRS sALS patient scores and percent of MDSC were analyzed using the two-tailed Student's paired t test. Differences in survival times were computed using Kaplan–Meier survival statistics (log-rank-sum test; Number Cruncher Statistical Systems, Kaysville, UT). Disease progression studies were analyzed using a one-way analysis of variance (ANOVA) with repeated measures (StatView software, v5; SAS Institute Inc.). Differences between groups were analyzed using a two-way ANOVA (JMP software, v5; SAS Institute, Cary, NC). Data from mouse studies are shown as mean±standard error (SEM).

## Supporting Information

Figure S1 **The levels of circulating MDSCs as a potential diagnostic marker for ALS.** Receiver operating characteristic (ROC) analysis curve indicating the prognostic discriminatory power of the percentage of MDSCs/PBMC in ALS patients, given a cutoff value of 2.28, compared with controls. **A.** Compared with all control subjects (n = 22), the overall area under the curve (AUC) (left panel) was 0.82 [95% confidence interval (CI): 0.698 to 0.945] with a sensitivity of 0.86 (95% CI: 0.67–0.96), and specificity of 0.73 (95% CI: 0.5–0.89). **B.** Compared with selected control subjects (n = 20), excluding those with severe immunosuppression (right panel), the AUC was 0.857 (95% CI: 0.745 to 0.97) with a sensitivity of 0.86 (95% CI: 0.67–0.96) and specificity of 0.8 (95% CI: 0.5–0.89).
(TIF)

Figure S2 **Relationship between the percentage of MDSCs and ALSFRS-R score over time.** **A**. Characteristics and main clinical features of sALS patients (n = 9) from two clinic visits at 3 month intervals. **B.** Fresh whole blood was incubated with a mixture of LIN, HLA DR, CD33 and CD45 mAbs. MDSC populations are shown in representative dot plots of sALS patients from two clinic visits. Numbers indicate the percentage of LIN^−/Low^, HLA-DR negative, CD33 positive cells of total PBMC according to CD45/SSC gating. **C.** The ALSFRS-R score was significantly lower (*p* = 0.05, paired T-test) in the second clinic visit compared with the first visit, while a clear trend to increased values in MDSC percentage was observed (*p* = 0.096, paired T-test).
(TIF)
