# Supplementary figures and images for: Excess Circulating Alternatively Activated Myeloid (M2) Cells Accelerate ALS Progression While Inhibiting Experimental Autoimmune Encephalomyelitis
Source: PLoS One. 2011 Nov 3;6(11):e26921. doi: 10.1371/journal.pone.0026921 (PMC3207825; doi:10.1371/journal.pone.0026921)

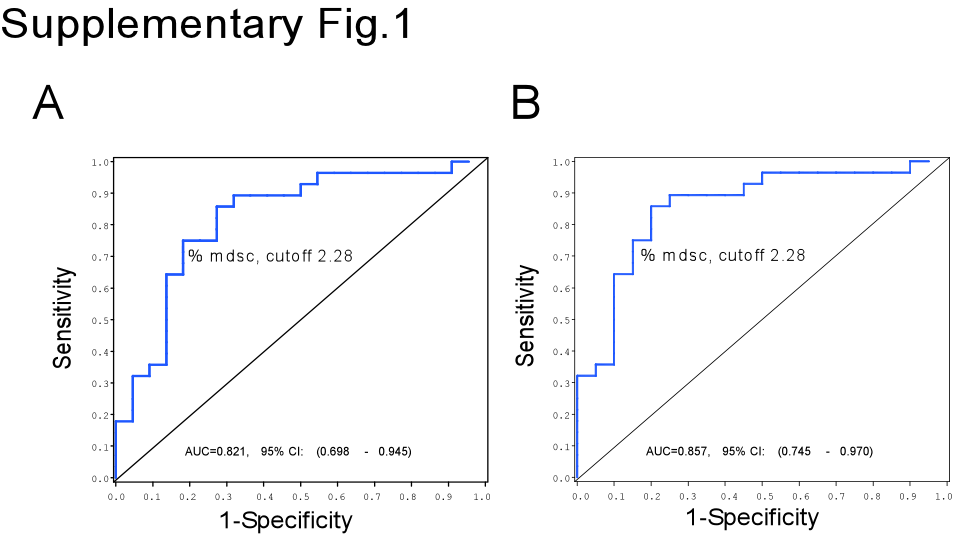

Supplement: Figure S1 — The levels of circulating MDSCs as a potential diagnostic marker for ALS. Receiver operating characteristic (ROC) analysis curve indicating the prognostic discriminatory power of the percentage of MDSCs/PBMC in ALS patients, given a cutoff value of 2.28, compared with controls. A. Compared with all control subjects (n = 22), the overall area under the curve (AUC) (left panel) was 0.82 [95% confidence interval (CI): 0.698 to 0.945] with a sensitivity of 0.86 (95% CI: 0.67–0.96), and specificity of 0.73 (95% CI: 0.5–0.89). B. Compared with selected control subjects (n = 20), excluding those with severe immunosuppression (right panel), the AUC was 0.857 (95% CI: 0.745 to 0.97) with a sensitivity of 0.86 (95% CI: 0.67–0.96) and specificity of 0.8 (95% CI: 0.5–0.89). (TIF) [file pone.0026921.s001.tif]

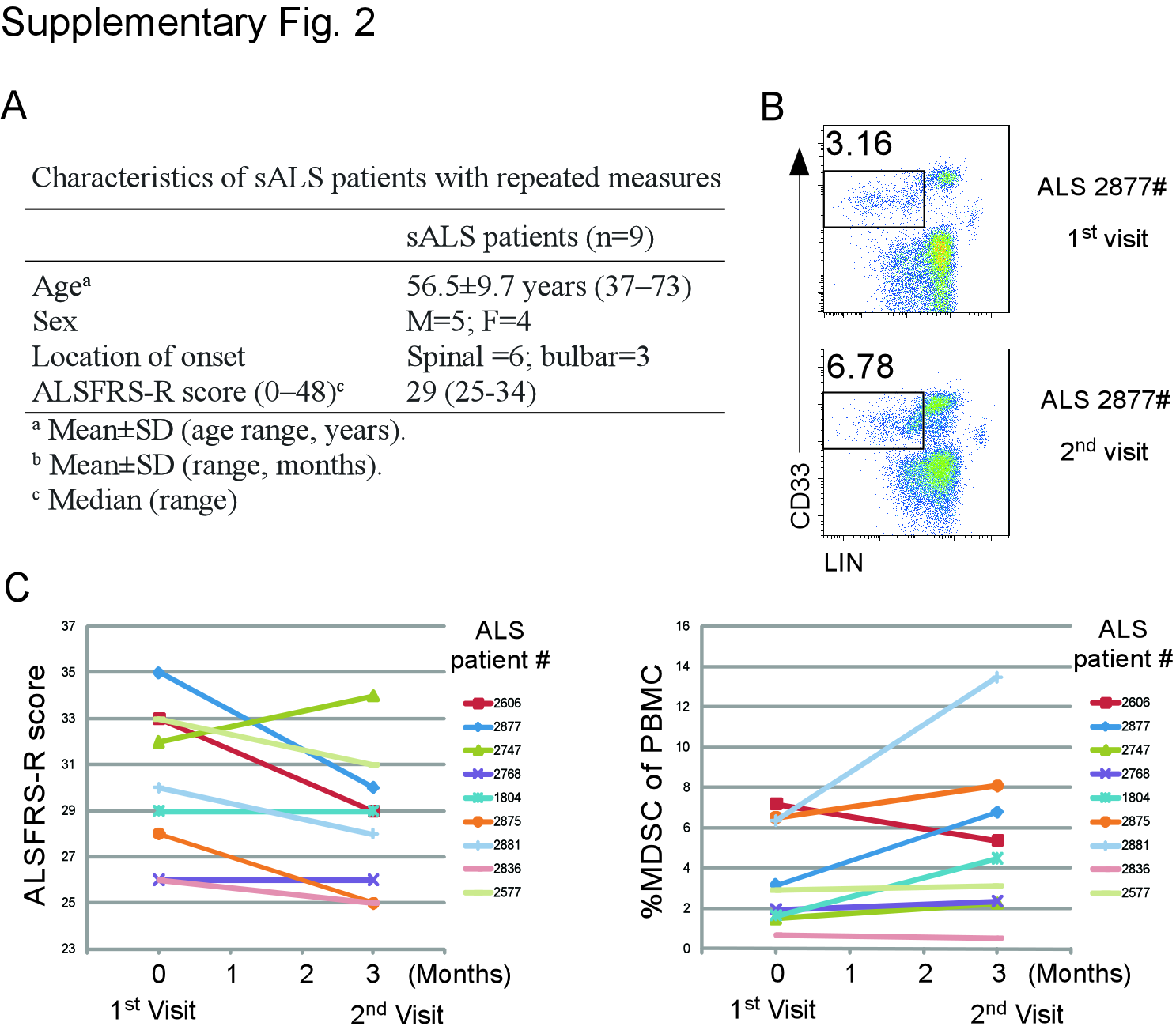

Supplement: Figure S2 — Relationship between the percentage of MDSCs and ALSFRS-R score over time. A. Characteristics and main clinical features of sALS patients (n = 9) from two clinic visits at 3 month intervals. B. Fresh whole blood was incubated with a mixture of LIN, HLA DR, CD33 and CD45 mAbs. MDSC populations are shown in representative dot plots of sALS patients from two clinic visits. Numbers indicate the percentage of LIN−/Low, HLA-DR negative, CD33 positive cells of total PBMC according to CD45/SSC gating. C. The ALSFRS-R score was significantly lower (p = 0.05, paired T-test) in the second clinic visit compared with the first visit, while a clear trend to increased values in MDSC percentage was observed (p = 0.096, paired T-test). (TIF) [file pone.0026921.s002.tif]
